# Improving post-injury follow-up survey response: incorporating automated modalities

**DOI:** 10.1186/s40621-024-00531-3

**Published:** 2024-09-05

**Authors:** Hannah Scheuer, Kelsey M. Conrick, Brianna Mills, Esther Solano, Saman Arbabi, Eileen M. Bulger, Danae Dotolo, Christopher St. Vil, Monica S. Vavilala, Ali Rowhani-Rahbar, Megan Moore

**Affiliations:** 1 School of Social Work, University of Washington, Seattle, WA USA; 2 Harborview Injury Prevention and Research Center, University of Washington, Seattle, USA; 3 Department of Epidemiology, University of Washington, Seattle, USA; 4 School of Social Work, University at Buffalo, Buffalo, USA; 5 Department of Trauma Surgery, Harborview Medical Center, Seattle, USA; 6 Department of Pulmonary, Critical Care, and Sleep Medicine, University of Washington, Seattle, USA; 7 Department of Anesthesiology and Pain Medicine, University of Washington, Seattle, USA

**Keywords:** Health equity, Disparities, Trauma registry, Injury

## Abstract

**Background:**

Incorporating post-discharge data into trauma registries would allow for better research on patient outcomes, including disparities in outcomes. This pilot study tested a follow-up data collection process to be incorporated into existing trauma care systems, prioritizing low-cost automated response modalities.

**Methods:**

This investigation was part of a larger study that consisted of two protocols with two distinct cohorts of participants who experienced traumatic injury. Participants in both protocols were asked to provide phone, email, text, and mail contact information to complete follow-up surveys assessing patient-reported outcomes six months after injury. To increase follow-up response rates between protocol 1 and protocol 2, the study team modified the contact procedures for the protocol 2 cohort. Frequency distributions were utilized to report the frequency of follow-up response modalities and overall response rates in both protocols.

**Results:**

A total of 178 individuals responded to the 6-month follow-up survey: 88 in protocol 1 and 90 in protocol 2. After implementing new follow-up contact procedures in protocol 2 that relied more heavily on the use of automated modalities (e.g., email and text messages), the response rate increased by 17.9 percentage points. The primary response modality shifted from phone (72.7%) in protocol 1 to the combination of email (47.8%) and text (14.4%) in protocol 2.

**Conclusions:**

Results from this investigation suggest that follow-up data can feasibly be collected from trauma patients. Use of automated follow-up methods holds promise to expand longitudinal data in the national trauma registry and broaden the understanding of disparities in patient experiences.

**Supplementary Information:**

The online version contains supplementary material available at 10.1186/s40621-024-00531-3.

## Background

Trauma patient follow-up in the United States (US) has challenged researchers and trauma care teams for decades (Aaland et al. 2012). Data from US trauma patients’ medical records are abstracted into local, state, and national trauma registries to guide intervention and prevention efforts, direct quality improvement, track patient health trajectories and outcomes, and examine the ways in which provider and systemic factors may contribute to disparities in outcomes (Moore et al. 2019; Surgeons ACo. 2022). However, current gaps in this system limit researchers’ ability to identify groups at highest risk for disparities in functional recovery. Prior research has established the need for a data collection system to collect long-term follow-up data from patients after injury (Graves et al. 2017; Herrera-Escobar et al. 2022; Conrick et al. 2022). Despite this critical need, and the successful implementation of follow-up procedures in many other countries (Gabbe et al. 2010; Vliet et al. 2019), post-discharge follow-up in the US has remained a considerable challenge. Moreover, significant disparities exist in trauma care follow-up, with persons of color and those with higher poverty and lower education levels consistently presenting lower follow-up rates (Leukhardt et al. 2010). Specific mechanisms driving these disparities are largely understudied (Moore et al. 2019; Conrick et al. 2022), pointing to a demonstrated need for post-trauma follow-up data that may support trauma care providers in understanding disparities and identifying targets for future intervention (Conrick et al. 2022). Given these gaps in data collection, there is a significant need for a feasible, cost-effective, and culturally resonant follow-up data collection process to be incorporated into existing trauma care systems.

The COVID-19 pandemic has presented additional challenges to reaching patients after discharge and a unique opportunity for swift adaptations to healthcare service delivery. With pandemic-related restrictions on in-person contact and post-discharge follow-up, data collection has been and continues to be adapted to virtual modalities including text and email. These virtual healthcare services that were rapidly implemented with the onset of COVID-19 may ultimately serve to reduce disparities in access to care (Mann et al. 2020). Prior research with hard-to-reach trauma populations, including patients with increased environmental instability and/or co-occurring mental health and substance use disorders, has consisted of persistent and intensive follow-up outreach efforts, with limited success (Lyons et al. 2021). These efforts are not sustainable long-term or feasible in the context of the COVID-19 pandemic, and there is a need for follow-up methods that reduce the burden on research and hospital staff. National trauma care experts have expressed an interest and need to find a feasible, cost-effective, and low-burden methods like emails, text messages, or automated phone calls, to follow-up with trauma patients (Conrick et al. 2022). Moreover, trauma registry experts have noted that due to limited resources, calling patients individually is not feasible for long-term follow-up, and an automated system is needed (Conrick et al. 2022).

The current study assessed and compared post-injury survey response rates across two distinct protocols utilizing different outreach modalities including mail, telephone, text, and email to determine feasibility of collecting patient-reported follow-up data from trauma patients 6 months after hospital admission, as well as most efficient data collection modalities.

## Methods

### Design

This study was part of a larger prospective cohort study conducted at a level-1 trauma center in the Pacific Northwest. Interviews were conducted with *n* = 245 racially and ethnically diverse trauma patients to develop a culturally resonant data collection system for equity-related measures. Six months after enrollment, study participants were contacted to complete the PROMIS-29 (Hays et al. 2018), a follow-up health-related quality of life survey measure. To test the feasibility of an automated system, researchers used Twilio, a low-cost ($0.007 per outgoing message) module within the Research Electronic Data Capture (REDCap, versions 9.4.2–10.2.1) survey platform (Harris et al. 2009, 2019) that automatically sends text messages or emails at specified intervals. All participants were given a $10 gift card upon completion of the interview. Study procedures were approved by the University Institutional Review Board (IRB). The STROBE guidelines were used to ensure proper reporting of methods, results, and discussion (see Supplemental Content 1). More details on the original study are available in Supplemental Content 2.

### Eligibility

Potentially eligible patients were identified via electronic medical record review. Inclusion criteria were: 18 years or older, admitted for at least 24 h with a physical injury (preliminary ICD-10 codes S00-T88 or V00-Y99), and had the ability to consent and interview in English or Spanish. Patients were excluded if they had a burn injury, were under law enforcement supervision, were under care for a secondary complication to a prior injury sustained more than 2 weeks prior to screening, or were unable to consent to participate in research as judged by nursing staff (e.g., those with cognitive impairments).

### Recruitment and procedure

**Fig. 1:**
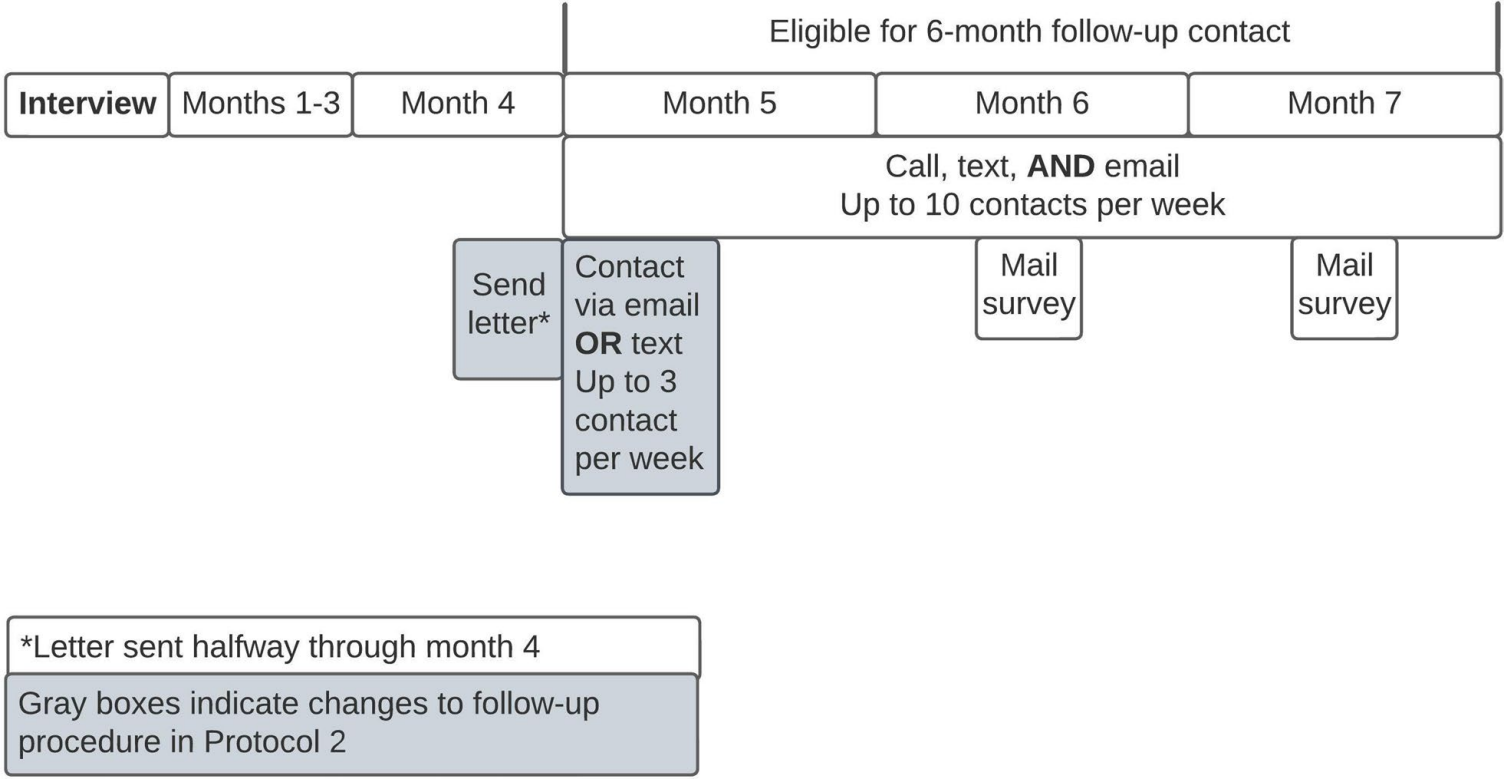
6-Month Follow-up Contact Protocols

We used a purposive sampling strategy to ensure a racially and ethnically diverse sample. The recruitment process was modified because of changes to study site procedures due to the COVID-19 pandemic (see Supplemental Content 3). *Protocol 1.* Patients in protocol 1 were enrolled from 6/24/19 to 3/6/2020. These patients were approached by the bedside, provided with the purpose of the study, consented, and completed a 25–40-min audio-recorded interview. Participants (*N* = 136) were asked to provide their phone, email, and mailing address, as well as additional contact information for two friends or relatives, their work, and their health clinic. Six months later, patients were contacted up to 10 times per week via multiple simultaneous outreach methods including phone, text, e-mail, and mail. They were asked to participate in a 10-min interview during which they completed the PROMIS-29, a short form assessment containing one pain intensity question and four items from seven domains: depression, anxiety, physical function, pain interference, fatigue, sleep disturbance, and ability to participate in social roles and activities (Hays et al. 2018). Participants were eligible to complete the follow-up survey from 5 to 7 months after initial interview. In an effort to yield high follow-up response rates (Lyons et al. 2021), research staff prioritized contacting patients for follow-up in as many ways possible and engaged in phone, text message, and email outreach concurrently (Fig. 1). Paper surveys with return postage were sent to participants who had not responded during second week of months 6 and 7 after interview. *Protocol 2.* Due to restrictions on in-person contact stemming from the COVID-19 pandemic, recruitment for protocol 2 patients was conducted remotely from 4/15/2020–8/30/2020. Potentially eligible patients were called while they were admitted to the hospital and offered the option to participate in the interview then or be contacted again post-discharge. Potentially eligible patients who had already been discharged from the hospital were contacted by phone up to 10 times in the two weeks after discharge. Once they had consented to participation and completed the interview, participants were asked to provide the same contact information as protocol 1 participants.

To increase follow-up survey response rates and further test the feasibility of low-cost automated contact options (i.e., text and email sent through Twilio), the study team implemented a new 6-month follow-up contact procedure during the COVID-19 pandemic (Fig. 1). Prior research demonstrates that sending a letter before asking participants to complete follow-up surveys increased telephone survey response rate by 11% (Leeuw et al. 2007). In line with this evidence, the investigative team sent a letter by mail to participants (*N* = 109) two weeks prior to them being eligible for 6-month follow-up; participants were eligible to complete the survey 5–7 months after initial interview. Additionally, as with prior literature has demonstrated that simultaneously offering multiple response modalities delays decision-making and decreases response rates (Medway and Fulton 2012), participants were only offered one modality (either text or email) for the first 1–2 weeks of eligibility (Fig. 1). Paper surveys with return postage were sent to participants who had not responded during second week of months 6 and 7 after interview.

### Data analysis

Once participants completed the follow-up survey, research staff recorded the modality by which the participant responded. Descriptive statistics, calculated with STATA version 14, were used to summarize participant demographics, as well as report and compare the frequency of follow-up response by modality and overall response rates across the two protocols.

## Results

A total of 178 individuals out of 245 eligible participants responded to the 6-month follow-up survey: 88 using protocol 1 and 90 using protocol 2 (Table 1). Overall response rate was 64.7% for protocol 1 and 82.6% for protocol 2. Most (*n* = 124; 69.6%) self-identified as persons of color.

**Table 1:** Demographics of participants who responded to 6-month follow-up survey according to self-report (where indicated) and medical record

	Protocol 1 *n* = 88	Protocol 2 *n* = 90	Total *n* = 178
Self-reported race* [N (%)]			
American Indian or Alaska native	11 (12.5)	7 (7.8)	18 (10.1)
Asian	8 (9.1)	4 (4.4)	12 (6.7)
Black	20 (22.7)	14 (15.6)	34 (19.1)
Hispanic or Latin(x)	30 (34.1)	24 (26.7)	54 (30.3)
Native Hawaiian or Pacific Islander	6 (6.8)	–^^^	–^^^
White	27 (30.7)	46 (51.1)	73 (41.0)
*Ethnicity [N (%)]*			
Hispanic or Latino	30 (34.1)	22 (24.4)	52 (29.2)
Not Hispanic or Latino	58 (65.9)	68 (75.6)	126 (70.8)
*Sex [N (%)]*			
Female	27 (30.7)	28 (31.1)	55 (30.9)
Male	61 (69.3)	62 (68.9)	123 (69.1)
*Language [N (%)]*			
English	74 (84.1)	79 (87.8)	153 (86.0)
Mandarin	–^^^	–^^^	–^^^
Spanish or Mixteco Alto	12 (13.6)	11 (12.2)	23 (12.9)
Other	–^^^	–^^^	–^^^
*Interview Language [N (%)]*			
English	75 (85.2)	76 (84.4)	151 (84.8)
Spanish	13 (14.8)	14 (15.6)	27 (15.2)
Age (mean, standard deviation [SD])	47.6 (17.1)	41.7 (15.7)	44.6 (16.6)

*Race categories are not mutually exclusive and some participants self-identified as more than one race

^Cell sizes < 5 not shown for participant confidentiality

Overall, after implementing new follow-up contact procedures, the response rate by any modality increased by 17.9 percentage points from protocol 1 (64.7%) to protocol 2 (82.6%) (Table 2). In protocol 1, the primary response modality was by phone (72.7%), and email and text only made up a combined 12.5% of responses. In contrast, the combination of email (47.8%) and text (14.4%) made up more than half of the responses during use of protocol 2 (62.2%), with phone accounting for only 35.6%, suggesting that automated and efficient modalities (e.g., email and text messages) are feasible methods for trauma care follow-up data collection. Mail responses also decreased between the two phases (14.8 to 2.2%).

**Table 2:** Follow-up response modality by Protocol

Modality	Protocol 1 (*n* = 88)		Protocol 2 (*n* = 90)	
	Count	Percent (%)	Count	Percent (%)
Phone	64	72.7	32	35.6
Mail	13	14.8	2	2.2
Email	8	9.1	43	47.8
Text	3	3.4	13	14.4

## Discussion

The current study is an innovative pilot of the feasibility of collecting follow-up data from racially and ethnically diverse patients in the US who have experienced injury via cost-efficient and low-burden automated modalities including text and e-mail. Findings from this investigation reveal that the implementation of a follow-up outreach procedure that included sending a letter two weeks prior to requests for follow-up completion and reducing the number of options for initial contact was associated with an increase in response rate. Furthermore, implementation of this purposeful modification in outreach procedure changed the most common response modality from phone, a higher cost and higher administrator burden modality, to a combination of email and text message, which may be automated and therefore more cost-efficient alternatives. Taken together, our results represent a first step towards development of a feasible automated and cost-efficient data collection process for follow-up data from trauma patients.

This investigation identifies key areas for future research. First, this investigation is US centric and does not describe successful post-discharge follow-up procedures that have been implemented in other countries (Gabbe et al. 2010; Vliet et al. 2019). Future research should be informed by lessons learned from successful post-discharge follow-up efforts implemented in other countries. While this study did not assess patient preference for follow-up outreach modality, recent evidence suggests that patients who speak a language other than English may have specific preferences for non-phone outreach (Gabbe et al. 2022). Differences in modality of response may also influence the completeness of data collected (Jojczuk et al. 2023). Assessing patient preference should be a priority in future studies.

While this study offers important insights into follow-up with hard-to-reach trauma populations, limitations must be noted. Results from this investigation could be impacted by selection bias. The trauma population fundamentally shifted during COVID-19, with fewer overall trauma admissions and pronounced differences in traumatic injury mechanisms (Jojczuk et al. 2023). For this study, COVID-19 also necessitated a shift to solely remote recruitment procedures for some of the participants. With remote recruitment, these participants had to answer the phone in order to be enrolled in the study, whereas protocol 1 participants were approached and enrolled by the bedside. This difference in recruitment strategies coupled with an overall change in the trauma population during COVID-19, could suggest fundamental differences between cohorts that may have impacted response rates. There were also several changes made between the two protocols and investigators cannot be sure whether improvement in response rate is due to one change or a combination of the changes. Moreover, although the results of this investigation speak to the promise of incorporating low-burden and cost-effective automated follow-up modalities, it must be noted that traditional mailers were utilized to achieve high follow-up response rates. The use of traditional mailers is not automated and may in some instances be less-cost effective, therefore future research should identify alternatives to traditional mailers that will facilitate post-trauma follow-up in diverse populations. Additionally, future research should include a detailed cost analysis to ensure that costs of implementing this follow-up protocol are sustainable. Finally, all study participants were recruited from one level-1 trauma center located in the northwest and specific demographic information that has been linked to disparities in trauma care follow-up and post-trauma outcomes (e.g., education level and socioeconomic status) were not collected for this sample. Further research is needed to ensure findings are generalizable on a national scale and include more robust demographic variables that may provide additional insight into the disparities that exist in trauma-care follow up.

Recent scholarship has established expert consensus on outcome measures for data collection from trauma patients (Herrera-Escobar et al. 2022). However, there is a demonstrated and significant need for the implementation of a feasible and cost-effective follow-up data collection process to be incorporated within existing trauma care systems. Trauma registry experts have identified that this system should prioritize automated data collection processes to increase willingness of trauma centers to implement procedures for long-term follow-up with trauma patients (Conrick et al. 2022). This investigation provides evidence that the implementation of a cost-efficient and automated data collection process may be both feasible and effective for following up with US trauma patients and ultimately expanding the national trauma registry to include data crucial for promoting long-term positive outcomes.

## Supplementary Information

Supplementary file 1: STROBE checklist.

Supplementary file 2: Flow chart of patient eligibility, contact, interviews, and follow-up procedures for parent study and current study

Supplementary file 3: Similarities and Differences Between Protocol 1 and Protocol 2.

## Data Availability

No datasets were generated or analysed during the current study.
