# Supplementary figures and images for: Improving post-injury follow-up survey response: incorporating automated modalities
Source: Inj Epidemiol. 2024 Sep 5;11:46. doi: 10.1186/s40621-024-00531-3 (PMC11375847; doi:10.1186/s40621-024-00531-3)

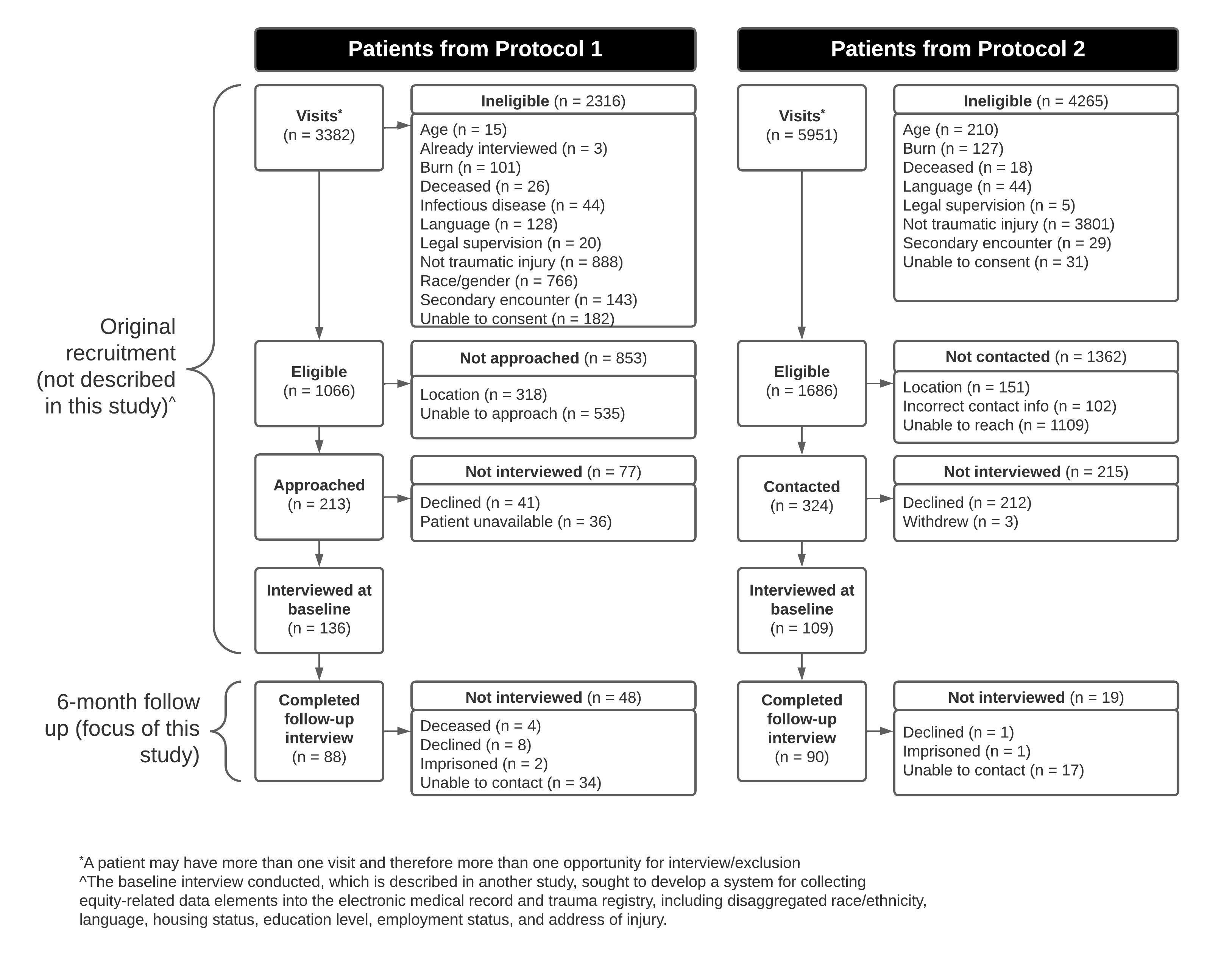

Supplement: Supplementary file 2 — Flow chart of patient eligibility, contact, interviews, and follow-up procedures for parent study and current study [file 40621_2024_531_MOESM2_ESM.jpeg]
