# Supplementary material for: Improving post-injury follow-up survey response: incorporating automated modalities
Source: Inj Epidemiol. 2024 Sep 5;11:46. doi: 10.1186/s40621-024-00531-3 (PMC11375847; doi:10.1186/s40621-024-00531-3)
Supplement: Supplementary file 3 — Similarities and Differences Between Protocol 1 and Protocol 2. [file 40621_2024_531_MOESM3_ESM.docx]

**Supplemental Content 2:** Similarities and Differences Between Protocol 1 and Protocol 2

|  | **Protocol 1** | **Protocol 2** |
| --- | --- | --- |
| Dates of baseline enrollment | June 24, 2019 – March 6, 2020 | April 15, 2020 – August 30, 2020 |
| Baseline enrollment location | Inpatient bedside | Phone call |
| Follow-up approach | Contact via as many modalities as possible concurrently | Prioritize lowest-cost, automated modalities first |
| Follow-up protocol | 1) Up to 10 contacts from all electronic modalities concurrently (phone, text, and email)  2) Mailed survey week 2 of months 6 and 7 | 1) Letter sent 2 weeks before eligibility  2) Initially offered only one automated response modality (text or email, based on participant preference) for the first 1-2 weeks of eligibility (Fig 1)  3) Mailed survey week 2 of months 6 and 7 |
